# Chromosome Inequality: Causes and Consequences of Non-Random Segregation Errors in Mitosis and Meiosis

**DOI:** 10.3390/cells11223564

**Published:** 2022-11-11

**Authors:** Sjoerd J. Klaasen, Geert J. P. L. Kops

**Affiliations:** 1 Hubrecht Institute—KNAW (Royal Netherlands Academy of Arts and Sciences) and University Medical Centre Utrecht, 3584 CT Utrecht, The Netherlands; 2 Oncode Institute, 3521 AL Utrecht, The Netherlands

**Keywords:** mitosis, meiosis, chromosomal instability, non-random segregation errors, cancer, embryo, development, aneuploidy

## Abstract

Aneuploidy is a hallmark of cancer and a major cause of miscarriages in humans. It is caused by chromosome segregation errors during cell divisions. Evidence is mounting that the probability of specific chromosomes undergoing a segregation error is non-random. In other words, some chromosomes have a higher chance of contributing to aneuploid karyotypes than others. This could have important implications for the origins of recurrent aneuploidy patterns in cancer and developing embryos. Here, we review recent progress in understanding the prevalence and causes of non-random chromosome segregation errors in mammalian mitosis and meiosis. We evaluate its potential impact on cancer and human reproduction and discuss possible research avenues.

## 1. Introduction

At every mitotic or meiotic division, cells must accurately segregate their chromosomes into two new daughter cells. Failure to do so leads to aneuploidy, which is defined here as a copy number of deviations from (a multiple of) the haploid set of whole or large parts of chromosomes. Aneuploidy is one of the most striking genomic abnormalities in cancer and is surprisingly common during embryonic development. Depending on the cell type, normal cells in vitro missegregate chromosomes between 0.5 and 5.0 times per 100 cell divisions [1,2,3]. These low segregation error frequencies are supported by the observation that 2.2–4.0% of cells in healthy human liver, brain, and skin are aneuploid [4]; however, neurons in the brain have also been reported to be much more aneuploid [5]. Certain in vitro cancer cell lines missegregate chromosomes at orders of magnitude higher than healthy cells; these are highly aneuploid and display karyotype heterogeneity [1,3,6,7]. Aneuploidy is also surprisingly common during human embryonic development: around 31% of embryos analyzed after in vitro fertilization possess aneuploidies from segregation errors made during meiosis, while 74% of embryos possess mosaic aneuploidies from missegregations which occur during the first few mitotic divisions after fertilization [8]. Because aneuploid cells are selected against, the aneuploidy percentages rapidly decline when more developed embryos are assessed [9,10]. Nevertheless, certain trisomies, such as those of chromosome 13, 18, or 21, are compatible with life.

Why is aneuploidy so common in cancer, but so uncommon in healthy adult tissue? First, cancer cells more frequently missegregate chromosomes during cell divisions; this is a phenotype known as chromosomal instability (CIN) [1,3]. CIN is thought to provide cancer cells with various advantages; indeed, inducing CIN can initiate cancer and promote metastasis [11,12,13]. Despite much effort, it remains unclear what the molecular causes for this are, and there could be many [14]. Second, cancer cells have evolved to deal with the problematic consequences of aneuploidy. Expression levels of genes on a given chromosome are scaled with their copy number states [15,16,17]. This leads to an array of stresses of, for example, proteotoxic or genotoxic origin [15,18,19,20,21], often leading to a p53-dependent arrest [15,22]. Because of this, non-transformed cells with experimentally induced aneuploidies have a proliferative disadvantage over their diploid counterparts [15,17]. However, many cancers have evolved mechanisms to cope with these disadvantages. These include mutations in the p53 pathway, whole-genome doubling, which buffers against gene dosage changes, increased expression of pathways responsible for resolving the stresses, and gene dosage compensation of stress-inducing proteins [16,23,24,25].

**Figure 1:**
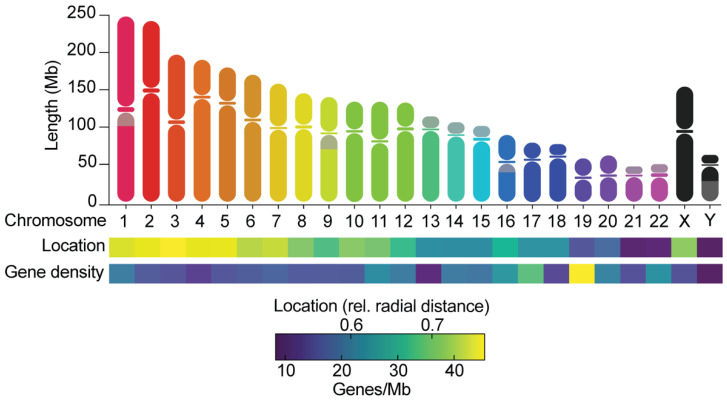
Chromosomes differ in many ways. The graph shows the length of chromosome arms and the location of centromeres, the relative radial distances of chromosomes towards the center of the nucleus [37], and the gene densities, as calculated from Ensembl (GRCh38.p13). Grey areas on chromosomes indicate the presence of large heterochromatin blocks. Chromosomes can be metacentric (centromere is near the middle of the chromosome), submetacentric (centromere is more towards one side of the chromosome), or acrocentric (centromere is near the end of a chromosome).

Whole- and arm-level aneuploidy profiles of cancers are similar within tissue types but different between tissues [23,26,27]. For example, colorectal cancers typically gain chromosomes 7, 8, and 13, but lose chromosome 18, while squamous cancers lose chromosome arm 3p but gain 3q [23,28]. One type of structural aneuploidy, called chromothripsis-like patterns, preferentially resides on certain chromosomes [29,30,31]. Chromothripsis occurs due to the rupture of micronuclei, which originate from missegregation events, and is characterized by extensive genomic rearrangements and oscillating copy number patterns restricted to a single chromosome [32,33]. Likewise, only certain chromosomes can be found at altered copy number states at late stages of human embryo development (gain of chromosome 13, 18, 21, and X and loss of the X or Y chromosome) [34]. How do cells develop such recurrent aneuploidy patterns? One likely explanation is selection. In cancer, tissue-specific aneuploidies can achieve amplifications of oncogenes and loss of tumor suppressors that result in an increased fitness of cells from that particular tissue [35,36]. Similarly, during embryogenesis, only cells with aneuploidies of small chromosomes are able to propagate, since these contain relatively few genes and thus suffer less from cellular stresses. However, it is becoming clear that another factor may have important contributions: the non-random missegregation frequencies of specific chromosomes that impact which chromosomes are more or less likely to be lost or gained. Chromosomes differ in various ways (Figure 1) and several of these can impact their behavior in mitosis. This review focuses on recent progress in understanding non-random chromosome segregation errors in mammalian cells. We discuss the current evidence for non-random chromosome segregation errors in mitosis and meiosis and its various proposed causes. Furthermore, we will highlight the importance of non-random segregation errors in tumor evolution and human reproduction.

## 2. Evidence for Non-Random Chromosome Segregation Error Frequencies

### 2.1. In Mitosis

The first observations of non-random segregation errors came from micronucleus assays, which measure the genotoxicity of compounds by quantifying the number of micronuclei in cell culture [38]. Micronuclei in lymphocytes treated with different clastogenic and aneugenic agents were shown to have various non-random chromosomal contents, depending on the agents used or the setup of the experiments (for an extensive list, see Norppa et al. (2003)) [38]. Although certain chromosomes may be more prone to becoming micronucleated [39,40], micronuclei originate from recent segregation error events and are therefore regarded as a proxy for recently missegregated chromosomes. As such, non-random micronuclear content in these assays is indicative of non-random segregation errors upon treatment with the toxic agents. Interestingly, chromosomes that end up in micronuclei have a higher probability of improperly segregating in the following mitoses [41,42]. Around half of micronuclei undergo membrane rupture [42], causing defective replication, inadequate nuclear protein import, epigenetic rewiring, exposure to cytosolic proteins [43,44,45], and missegregations during the next cell cycle [41,42]. Therefore, micronuclei catalyze the formation of chromosome-specific supernumerary and complex aneuploidies.

In recent years, advances in single-cell technologies such as fluorescence in situ hybridization (FISH) and single-cell DNA sequencing have allowed researchers to assess segregation error rates per chromosome by looking at the complete chromosome content of cells following disrupted mitosis. Release from a nocodazole-induced 8 h mitotic arrest specifically increased the missegregation frequencies of chromosomes 1 and 2 up to 11-fold in human cells [46]. A similar treatment in rat kangaroo kidney epithelial (PtK1) cells caused non-disjunction for six chromosomes, but less so for chromosomes 1 and 5 [47]. A release from monastrol, an Eg5 kinesin inhibitor, enriched for anaphase lagging of chromosomes 1 and 2 in human cells, chromosome 4 in PtK1 cells, and chromosome 3 + X in Indian Muntjac cells [47,48]. Compromising attachment error correction and the spindle assembly checkpoint (SAC) by inhibiting the kinase Mps1 revealed a high missegregation frequency of larger chromosomes with a difference up to threefold in human cells and of chromosome 3 + X in Indian Muntjac cells [33,48,49]. Larger chromosomes were also found to missegregate more frequently in human cells when attachment error correction was compromised by inhibiting Aurora B kinase, when microtubules were stabilized by low concentrations of taxol, or when chromosome congression was compromised by inhibition of the mitotic kinesin CENP-E [49,50]. Interestingly, generating DNA damage in human cells during the interphase, using ionizing radiation or by causing replication stress, specifically elevated structural rearrangements and micronuclear incorporation of larger chromosomes as well [33,51,52]. Lastly, loss of endogenous CENP-A in human cells increased the segregation error frequency of chromosomes 6, X, and Y [53,54]. In conclusion, non-random segregation errors are frequently observed when chromosomal instability is experimentally induced.

Can non-random missegregation events also be observed under more natural conditions? Small (<2.7 Mb) human artificial chromosomes in HT1080 cells were missegregated and were lost up to five times more often than their larger natural and artificial counterparts [55]. In human embryos, segregation errors during the first few divisions are frequent. Preimplantation genetic testing on single cells of 3-day-old human embryos showed that the frequency at which a given chromosome was found as aneuploid correlated with its size [10]. Likewise, in cancer cells, larger chromosomes were preferentially entrapped in the micronuclei of glioblastoma, colorectal cancer, and cervical cancer cell lines [49,56], and other biases were observed in three other cancer lines [49]. Furthermore, FISH for five chromosomes on anaphase figures in five glioma and one breast cancer cell line also found that larger chromosomes missegregated more often than smaller ones [57]. Taken together, non-random missegregations are also frequently observed in natural conditions of chromosomal instability and tend to correlate with chromosome size. 

### 2.2. In Meiosis

Male meiosis is relatively error-free, with around 1–5% of spermatozoa containing aneuploidies [58]. Single-cell DNA sequencing of 31,228 spermatozoa from 20 healthy donors established that the smaller acrocentric chromosomes and the sex chromosomes were ~2–6-fold more likely to be aneuploid, which was in line with FISH studies on spermatozoa and testicular biopsies [58,59,60]. Unlike male meiosis, female meiosis is notoriously erroneous and is therefore responsible for the large majority of meiotic-associated aneuploidies in embryos [58,61]. Observations using leftover oocytes from in vitro fertilization and in vitro and in vivo matured oocytes from biopsies of small antral follicles showed that the aneuploidy level is relatively high in females compared with males [58,62]. As in spermatocytes, sex or acrocentric chromosomes are often found to be aneuploid in oocytes [62]. Chromosome 16 is a notable exception, as it seems to missegregate the most often in female meiosis [63]. Oocytes in meiosis II of women below the age of 20 are ~1.7 times more likely to be aneuploid compared with those of women between the ages of 20 and 33 years (22% versus 37%) [62]. The errors mostly originate from non-disjunction events and are ~4 times more likely to affect large chromosomes compared with the acrocentric ones [62]. Oocytes of women above the age of 33 are 2.3 times more likely to be aneuploid compared with those of women between the ages of 20 and 33 years (24% versus 54%) and often involve premature separation of sister chromatids or reverse segregation of acrocentric or smaller chromosomes [62]. Similar aneuploidy landscapes were found in other studies as well [10,64,65,66,67,68]. 

## 3. Chromosome Segregation Mechanisms

**Figure 2:**
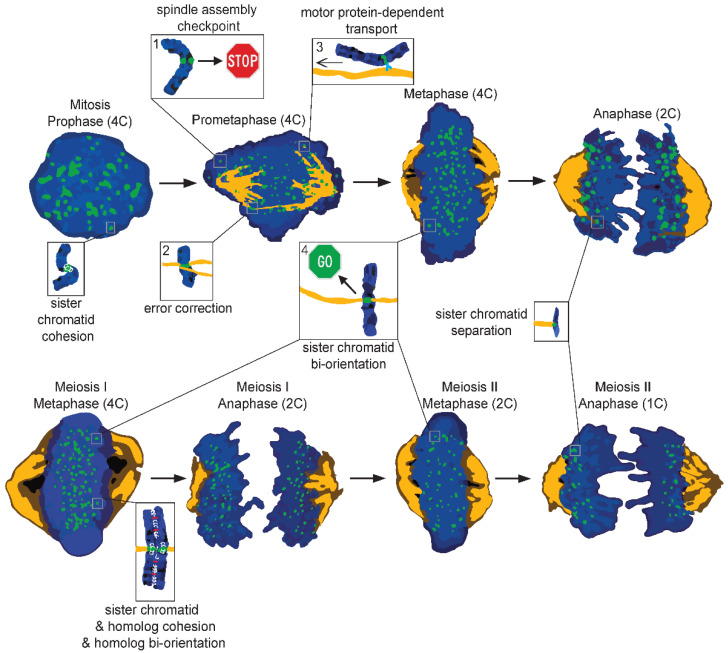
Cartoon illustrating different phases of mitosis (**top**) and meiosis (**bottom**). During mitosis, all the DNA is supposed to evenly split, creating two genetically identical daughter cells. Chromosomes (blue) are brought to the metaphase plate with the help of microtubules (orange) or motor proteins (light blue). During this phase, sister kinetochores (green) could be: (1) unattached, leading to the activation of the spindle assembly checkpoint (SAC); (2) erroneously attached, which is resolved by the error-correction machinery; (3) laterally attached, which allows for transport towards the metaphase plate; (4) end-on attached, which favors proper segregation. Only when all sister kinetochores are bioriented is the SAC silenced, causing the removal of centromeric cohesin (white) and the movement of sisters to opposite poles. Meiosis follows many of the same principles as mitosis, but differs as well. Instead of creating two genetically identical diploid daughter cells, meiosis generates four genetically different haploid cells. Furthermore, homologous chromosomes are not only affected by cohesin, but also by chiasmata (red), which are physical links created during genetic recombination. During meiosis, the homologous chromosomes are separated first followed by separation of the sisters. Zoom-ins in the figure highlight some of the mechanisms responsible for proper chromosome segregation; 4C, 2C, and 1C refer to DNA content.

In order to understand what causes non-random segregation errors of chromosomes, one must understand the basic principles governing chromosome segregation during cell division. We will therefore briefly discuss the general steps of mitosis and meiosis (Figure 2). 

Before cells can enter mitosis, all chromosomes must be replicated, and sister chromatids must be tightly bound to each other by a protein complex called cohesin [69]. The start of mitosis is defined by the start of the condensation of chromosomes. At the same time, cohesin is only removed from the chromosome arms, leading to the characteristic X shape. When condensation is completed, the nuclear envelope breaks down and highly dynamic tubulin proteins will polymerize into microtubules. These microtubules organize into a spindle, with two focused poles on opposite sides of the cell. In most cells, except oocytes of certain species such as humans [70], these poles are organized by centrosomes. Microtubules physically interact with chromosomes via kinetochores, which are multiprotein structures located on centromeres [71]. An intricate balance of pulling and pushing forces direct the movement of chromosomes to the metaphase plate, a process known as congression [72]. These movements are generated by the depolymerization or polymerization of microtubules, but also by microtubule motors such as CENP-E and dynein, which allow for the gliding of chromosomes on microtubules. Chromosomes located in the center of the nucleus almost instantaneously acquire the proper end-on attachments, meaning interactions of kinetochores with the +-ends of microtubules, and therefore achieve correct biorientation without much need for error-correction mechanisms [73]. However, the more peripheral chromosomes tend to first connect to the lattices of microtubules, and need the help of motor proteins and attachment error-correction mechanisms for conversion to end-on interactions and correct biorientation [72,74,75,76]. Only when all chromosomes have bioriented and congressed on the metaphase plate will anaphase commence: centromeric cohesin is released and sister chromatids are pulled to opposite sides. To make sure that anaphase does not start until all chromosomes are properly bioriented, cells employ the SAC pathway. This pathway, of which Mps1 is the master regulator [77], senses lack of interactions between kinetochores and microtubules and halts anaphase initiation accordingly [78]. Interestingly, the SAC is not activated when specific erroneous attachments are present, such as when one kinetochore is captured by both poles (merotely) or both kinetochores are captured by one pole (syntely) [79,80]. Instead, these attachment types are corrected by an error-correction pathway involving the kinase Aurora B [81,82,83,84]. 

Many of the mechanistic principles of chromosome segregation in mitosis also apply to meiosis [85]. Nevertheless, meiosis differs in several aspects. Meiosis occurs over two distinct steps: separation of homologs (meiosis I) and separation of sister chromatids (meiosis II, similar to mitosis). A defining step during meiosis I is the pairing of homologs through the recombination of chromosome arms into chiasmata. This process should take place at least once on every homolog as it is important for driving genetic variation and for keeping homologs together until anaphase I [86]. Probably the most striking feature of meiosis is its sexual dimorphism. In humans, female meiosis I starts in the germ cells during fetal development, is followed by a prolonged metaphase I arrest (also called dictyate arrest), and continues after ovulation to metaphase II until fertilization triggers the final steps. Male meiosis on the other hand takes place throughout adult life [86]. 

## 4. Mechanisms of Non-Random Chromosome Segregation Errors

### 4.1. Centromeric and Centromere-Proximal Features

Centromeres are vital for proper chromosome segregation since they assemble the kinetochore and are the site of sister chromatin cohesion. They have thus been implicated in multiple for non-random segregation error mechanisms. For example, treating cells with the microtubule poison nocodazole or with the kinesin inhibitor monastrol causes a mitotic arrest, leading to cohesion fatigue at the centromeres of specific chromosomes [46,87]. It is tempting to speculate that the frequently missegregating chromosomes have lower levels of centromeric cohesin molecules and that these are therefore more susceptible to cohesion fatigue, but this remains to be determined. Some cancers (e.g., glioblastoma) have mutations in cohesin-related genes and correcting these mutations in glioblastoma cell lines reduced CIN [88]. It would be of interest to establish whether the missegregation frequencies in these cells are similar to those with experimentally induced cohesion fatigue. 

Aside from a cohesion-related bias, the size of centromeres has also been implicated in affecting individual chromosome segregation error frequencies. In Indian Muntjac cells, missegregation frequencies after SAC inhibition and monastrol treatment were higher for chromosomes with larger centromeres [48]. Large centromeres bind more microtubules and are therefore perhaps more likely to make merotelic attachments, leading to segregation errors. Whether this phenomenon also takes place in other species is unclear. However, human kinetochores differ substantially in size, and computational modelling suggests that larger kinetochores may make more erroneous attachments [89,90]. Paradoxically, smaller centromeric domains may also increase missegregation probabilities. The centromeric proteins CENP-A and CENP-B are responsible for determining the location of kinetochores. As such, experimental CENP-A degradation forces cells to rely solely on CENP-B for assembling kinetochores, and this causes higher frequency of segregation errors for chromosomes whose centromeres had lower CENP-B levels, such as the Y chromosome [53,54]. Interestingly, a bias for Y chromosome micronucleation and copy number changes were observed in lymphocytes of aged versus younger individuals [91,92,93,94]. Ageing has been linked to reduced expression of the transcription factor FoxM1 [95], which, among other mitotic proteins, regulates expression of CENP-A [96]. Indeed, CENP-A protein levels decrease with age [97,98], raising the possibility that the increased micronucleation and copy number changes in aged individuals is caused by a mechanism similar to the one following experimental CENP-A degradation. In conclusion, centromeres were found to impact missegregation frequencies in multiple settings. 

### 4.2. Chromosome Size

As outlined in Section 2, depending on the mode of error induction, chromosome missegregation frequencies were found to correlate or anti-correlate with their size. Larger chromosomes are more often found near the periphery of the nucleus, which complicates their eventual biorientation (see Section 4.3). In addition, larger chromosomes have a higher likelihood of becoming damaged or containing elements which are prone to damage. For example, random DNA damage, such as ionizing radiation, induces structural rearrangements and the formation of micronuclei of larger chromosomes more often [33,52]. Furthermore, replication stress relatively frequently induces the formation of micronuclei containing larger chromosomes, because these are more likely to contain sequences sensitive to replication stress [6,51,99]. Replication stress is a common phenotype in cancer as it is induced by overexpression of various oncogenes [100]. 

While the nuclear position of larger chromosomes increases their chance of being missegregated, smaller chromosomes can also be at an increased risk. As discussed before, it is vital for every homolog in meiosis to have at least one chiasma. A lack of chiasmata (achiasmate) on a single pair of homologs allows them to drift apart and has been proposed to contribute to segregation errors in meiosis [101]. Chiasmata distribute evenly across DNA, meaning larger chromosomes, such as chromosome 1, have four chiasmata each on average, while smaller chromosomes such as Y chromosomes and acentric chromosomes only have one or two. An exception is the X chromosome, which is large but only has one chiasma [102,103]. Therefore, if every crossover has an equal chance of failing to reach maturity, larger chromosomes are less likely to be achiasmate, because other chiasmata compensate for a failed crossover event. Supporting this, smaller chromosomes in murine spermatocytes and human oocytes are more likely to be achiasmate, and missegregated chromosomes in human spermatozoa or oocytes have a decreased number of crossovers [104,105,106]. The female age-related increase in aneuploidy is also related to chromosome size. Cohesin is lost over time in oocytes and is not replenished [62,107,108,109]. Loss of cohesin abolishes chiasmata and creates gaps between homologs and sister chromatids, leading to erroneous kinetochore–microtubule attachments or even a complete loss of cohesion between chromosomes [110,111]. Progressive loss of cohesin between the homologs as well as the sister chromatids first affects chromosomes with shorter arms, because these have less overall cohesin. This also explains why oocytes of women 35–39 years of age are more likely to be aneuploid among smaller chromosomes than those of women aged 40 years and older [112,113]. Early age-related loss of cohesin in oocytes substantially decreases cohesion on smaller chromosomes, while further loss of cohesin in older women eventually also affects proper cohesion of larger chromosomes. In short, chromosome size may be an important cause for non-random segregation errors, especially in meiosis.

### 4.3. Chromosome Location

Chromosomes are not organized randomly in the interphase nucleus, which significantly impacts missegregation frequencies in multiple contexts. Larger chromosomes are frequently positioned in the periphery of the nucleus, while smaller chromosomes occupy a more central position [49,114]. Because of this, peripheral and thus larger chromosomes often end up behind the poles at the start of mitosis [74,115]. In turn, they need to travel a longer distance to the metaphase plate, are more likely to make non-amphitelic attachments (such as lateral or erroneous ones) [73,74,116], or might have difficulties crossing centrosomes. They thus need more time to become bioriented, in contrast to the almost instantaneously bioriented central chromosomes [117]. When cells were forced to prematurely enter anaphase due to SAC inhibition, these larger peripheral chromosomes missegregate more frequently [33,49]. This could be directly attributed to location differences rather than other chromosome-specific features: identical chromosomes have different missegregation frequencies when their locations are experimentally changed [49]. Larger chromosomes also missegregate more than smaller ones after inhibition of Aurora B, the master regulator of the error-correction pathway, and misaligned more often than smaller ones after altering microtubule dynamics or inhibiting CENP-E, a motor protein which is necessary for congression [49,50]. Do chromosome locations affect segregation error frequencies when chromosomal instability occurs naturally? Interestingly, micronuclear entrapment of chromosomes in cancer cells and aneuploidy frequencies during mitosis in human embryos and certain cancer cells correlate with chromosome size and location. In mice, SAC weakening occurs during the first error-prone mitotic divisions [118]. The non-random aneuploidies found in 3-day-old human embryos are therefore similar to the ones found in human somatic cells; their chromosome segregation fidelity can be experimentally compromised, and may thus be a consequence of chromosome locations in the nucleus. Additional causes for the aneuploidies are likely however, since segregation errors during the first division have been linked to poor clustering of the parental genomes after fertilization [119].

## 5. Consequences of Non-Random Chromosome Segregation Errors

### 5.1. Karyotype Evolution in Cancer

**Figure 3:**
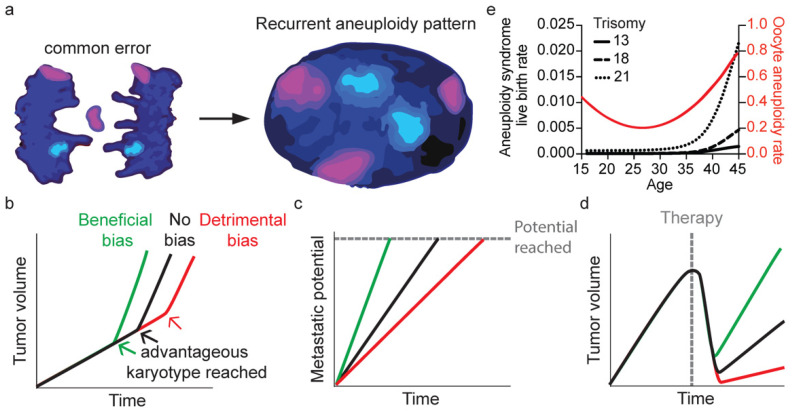
Hypothesized consequences of non-random segregation errors. (**a**) Frequently missegregating chromosomes gives rise to the recurrent aneuploidy pattern seen in cancer. Graphs depicting influence of non-random segregation errors on the speed by which a tumor grows (**b**), it reaches metastatic potential (**c**) or is able to grow out after therapy (**d**). (**e**) Graph showing the relationship between age and oocyte aneuploidy rate [62] or the aneuploidy syndrome live-birth rate [126].

Given the many observations of non-random segregation error frequencies for various chromosomes and the resulting non-random aneuploidy landscapes in cell populations following segregation error events, a major question is how this impacts genome evolution in cancer and human reproduction. Non-random segregation errors could, for example, contribute to the emergence of recurrent aneuploidy patterns observed in tumors (Figure 3a). Chromosome 1 is missegregated frequently in intestinal cancer cell lines and in error-induced intestinal organoids [49], which could impact the probability and timing by which the often-observed loss of 1p in colorectal adenocarcinomas occurs [120]. How much the non-random missegregation contributes to emergence of such patterns is unknown. Clonal outgrowth of cells that underwent the high-probability event of loss of chromosome 1 in this scenario could be due to neutral-drift dynamics or to positive selection. The former is not uncommon for single-nucleotide variants [121,122] and has been seen for copy number variations as well [123]. The question of which evolutionary dynamics underlie aneuploidy patterns in various cancers remains to be resolved. However, copy number variations occur more frequently on genomic regions containing cancer genes [35,36], which makes it likely that at least some aneuploidies are the result of positive selection. Although it has been investigated in only a limited number of settings in humans, this hypothesis is supported by the observation that the proliferation rates of diploid cells are higher than those of trisomic or monosomic cells in normal culturing conditions, and that specific aneuploidies are favored in certain challenging conditions [15,17,124,125]. This suggests that selection of aneuploidies is inherently non-neutral. Such observations were carried out among relatively normal cells; therefore, it remains to be determined whether this holds true for all chromosomes and chromosome combinations in different aneuploidy-tolerant non-transformed and transformed cell types, and under different selection pressures.

Another potential way by which non-random segregation errors can affect the evolution of karyotypes is by creating the initial aneuploidy landscapes on which selection acts, thereby impacting the time it takes to reach the recurrent aneuploidy patterns seen in cancer. Accordingly, non-random missegregations could impact multiple aspects of tumorigenesis, since specific recurrent aneuploidy patterns are associated with tumor proliferation, metastasis formation, and therapy resistance (Figure 3b–d) [127,128]. For example, clear-cell renal carcinoma metastases often lose chromosome arm 9p compared with the primary tumor [129]. A cell with this potentially beneficial aneuploid state can emerge in a population often if chromosome 9 relatively frequently missegregates. On the other hand, it would take substantially longer if chromosome 9 has a low missegregation probability. 

Non-random segregation errors may not only be important for the acquisition of aneuploidies but also for their maintenance. For example, a hypothetical cancer that obtained chromosome 5 trisomy because it provides a proliferative advantage may have reached this state quickly if chromosome 5 frequently missegregates, but will subsequently tend to lose it again for the same reason. This applies to CIN in cancer in general, and raises the important question of how CIN cells with favorable karyotypes stabilize that karyotype over time despite their CIN phenotype. Selection for reduced CIN rates or altered segregation error frequencies could in principle provide solutions to this. Unfortunately, experimental or theoretical examinations of the potential consequences of non-random segregation errors have yet to be performed. Genome evolution studies following the karyotypes of chromosomally unstable cells with different non-random missegregation frequencies or mathematical models of aneuploidy evolution that incorporate experimental data (such as known missegregation frequencies, cancer chromosomal instability levels, recurrent aneuploidy patterns, and the proliferative advantage of specific aneuploidies) should shed more light on these questions [130,131].

### 5.2. Aneuploid Live Births

In human embryos, non-random missegregations could potentially determine frequencies of chromosome-specific aneuploidies in live births. For example, larger chromosomes are aneuploid more frequently in the oocytes of younger women. This is expected to be incompatible with life, and indeed live aneuploidy births from young women are rare (Figure 3e) [62,126]. By contrast, smaller chromosomes are aneuploid more frequently in oocytes of older women. Small chromosome aneuploidies are less detrimental to cells, and indeed older women have a higher chance of live aneuploid births, which most commonly have trisomies (13, 18, 21) or monosomies (Y) of smaller chromosomes (Figure 3e). Although the majority of such aneuploidies are of meiotic origin, aneuploidies in live births can also be mosaic and thus of mitotic origin. Mitotic segregation errors in early development can have unique non-random segregation error patterns [8,10] and aneuploid cells from such errors tend to be outcompeted by diploid cells in the embryo [10,132]. Therefore, an interesting question remains: to what extent can aneuploidy patterns in live births and the subsequent age-dependent differences be attributed to potential non-random segregation errors of mitotic origin?

## 6. Concluding Remarks and Future Directions

As outlined above, increasing accumulating evidence indicates that segregation errors in mitosis and meiosis can be non-random, and some of the molecular explanations for this are emerging. These insights open up many fascinating new questions: Does compromising the different pathways implicated in chromosomal instability—such as hyperstable microtubule–kinetochore attachments, centrosomal defects/amplification, polyploidization, or cohesion fatigue [88,133,134,135,136,137]—cause non-random segregation errors? If so, what kinds? Does this differ between cell types (e.g., oocytes have a weak SAC and are acentrosomal) [138,139,140]? Do chromosomally unstable cancers experience non-random segregation errors? If so, can cancer types be distinguished by their bias? What are the consequences of non-random segregation errors for the evolution of karyotypes and therefore for the evolution of cancer? Large-scale single-cell sequencing research efforts, new mathematical models for chromosome copy number evolution, and in-depth molecular characterization of chromosome-specific behaviors during division of various cell types will shed light on the answers to these questions. 

## Data Availability

Not applicable.
